# A Hybrid Ionic Liquid–HPAM Flooding for Enhanced Oil Recovery: An Integrated Experimental and Numerical Study

**DOI:** 10.3390/polym18030359

**Published:** 2026-01-29

**Authors:** Mohammed A. Khamis, Omer A. Omer, Faisal S. Altawati, Mohammed A. Almobarky

**Affiliations:** 1 Department of Petroleum and Natural Gas Engineering, College of Engineering, King Saud University, Riyadh P.O. Box 800, Saudi Arabiammobarky@ksu.edu.sa (M.A.A.); 2 Department of Mechanical Engineering, College of Engineering, King Saud University, Riyadh P.O. Box 800, Saudi Arabia

**Keywords:** enhanced oil recovery, ionic liquid, HPAM, polymer flooding, core flooding, numerical simulation, high salinity, mobility control

## Abstract

Declining recovery factors from mature oil fields, coupled with the technical challenges of recovering residual oil under harsh reservoir conditions, necessitate the development of advanced enhanced oil recovery (EOR) techniques. While promising, chemical EOR often faces economic and technical hurdles in high-salinity, high-temperature environments where conventional polymers like hydrolyzed polyacrylamide (HPAM) degrade and fail. This study presents a comprehensive numerical investigation that addresses this critical industry challenge by applying a rigorously calibrated simulation framework to evaluate a novel hybrid EOR process that synergistically combines an ionic liquid (IL) with HPAM polymer. Utilizing core-flooding data from a prior study that employed the same Berea sandstone core plug and Saudi medium crude oil, supplemented by independently measured interfacial tension and contact angle data for the same chemical system, we built a core-scale model that was history-matched with RMSE < 2% OOIP. The calibrated polymer transport parameters—including a low adsorption capacity (~0.012 kg/kg-rock) and a high viscosity multiplier (4.5–5.0 at the injected concentration)—confirm favorable polymer propagation and effective in -situ mobility control. Using this validated model, we performed a systematic optimization of key process parameters, including IL slug size, HPAM concentration, salinity, temperature, and injection rate. Simulation results identify an optimal design: a 0.4 pore volume (PV) slug of IL (Ammoeng 102) reduces interfacial tension and shifts wettability toward water-wet, effectively mobilizing residual oil. This is followed by a tailored HPAM buffer in diluted formation brine (20% salinity, 500 ppm), which enhances recovery by up to 15% of the original oil in place (OOIP) over IL flooding alone by improving mobility control and enabling in-depth sweep. This excellent history match confirms the dual-displacement mechanism: microscopic oil mobilization by the IL, followed by macroscopic conformance improvement via HPAM-induced flow diversion. This integrated simulation-based approach not only validates the technical viability of the hybrid IL–HPAM flood but also delivers a predictive, field-scale-ready framework for heterogeneous reservoir systems. The work provides a robust strategy to unlock residual oil in such challenging reservoirs.

## 1. Introduction

A substantial portion of crude oil, often exceeding two-thirds of the original oil in place, remains trapped in reservoir pores following the cessation of primary and secondary recovery methods [1]. This significant residual oil saturation represents a vast untapped resource and its extraction is limited by complex reservoir rock and fluid properties, displacement mechanisms, and economic constraints [2]. Enhanced oil recovery (EOR) techniques have been developed to overcome these limitations, with chemical EOR (CEOR) strategies specifically designed to mitigate capillary forces, alter rock–fluid interactions, and improve both displacement efficiency and volumetric sweep coverage [3,4].

Among CEOR methods, polymer flooding using partially hydrolyzed polyacrylamide (HPAM) is one of the most widely deployed and studied processes [5,6]. The primary mechanism of polymer flooding is to increase the viscosity of the injected aqueous phase, thereby improving the mobility ratio between the displacing fluid and the resident oil. A favorable mobility ratio suppresses viscous fingering, delays breakthrough, and enhances macroscopic sweep efficiency, leading to a higher recovery factor [7,8]. Beyond this bulk viscosity effect, the success of a polymer flood is governed by a complex set of rheological and transport phenomena in porous media. The rheology of HPAM solutions is non-Newtonian, typically exhibiting shear-thinning behavior that is influenced by polymer concentration, molecular weight, and the salinity and hardness of the brine [9,10]. The in situ viscosity, which dictates the actual mobility control, is therefore a function of both the polymer’s intrinsic properties and the shear environment within the reservoir rock.

The transport of polymer through porous media is further complicated by key rock–fluid interactions, primarily adsorption and inaccessible pore volume (IPV). Adsorption of polymer molecules onto the rock surface leads to chemical retention, reducing the effective polymer concentration propagating through the reservoir [11,12]. This phenomenon is often described by an adsorption isotherm and can also lead to a reduction in water permeability, quantified by the residual resistance factor (RRF), which can improve sweep by diverting flow into unswept zones [13]. Conversely, IPV, which arises because large polymer molecules cannot access the entire pore space, causes the polymer front to travel faster than a conservative tracer, affecting the timing of the chemical flood [14]. Accurately characterizing these parameters—in situ rheology, adsorption, RRF, and IPV—is not merely academic; it is critical for predicting polymer injectivity, propagation, and ultimate recovery efficiency [15].

The design of the polymer component remains critical to field applicability. Extensive laboratory studies have established clear correlations between polymer molecular architecture and performance under HTHS conditions [16,17,18]. In particular, optimizing the HPAM concentration is essential to balance viscosity enhancement, injectivity, and economic feasibility. Accurate prediction of in situ behavior of polymers must account for mechanical degradation during propagation through porous media [19,20]. Recent advances in characterizing viscoelastic effects further suggest that polymers can enhance displacement efficiency beyond simple mobility control [21,22]—a factor implicitly captured in our simulation through calibrated transport parameters and validated against core-flood recovery data.

However, the performance of conventional HPAM is severely compromised under high-temperature, high-salinity (HTHS) conditions, which are prevalent in many of the world’s carbonate and deep sandstone reservoirs [23,24]. In these harsh environments, HPAM suffers from thermal degradation and hydrolysis of its acrylamide backbone, leading to irreversible loss of molecular weight and viscosity [25,26]. Furthermore, interactions with divalent cations such as Ca^2+^ and Mg^2+^ cause polymer chain coiling, precipitation, and a dramatic reduction in solubility and viscosifying power [27,28]. These limitations have spurred the search for more robust chemical agents and novel formulations.

Ionic liquids (ILs)—a class of organic salts with melting points below 100 °C—have emerged in the last decade as promising advanced materials for the oil and gas industry [29,30]. Defined by their negligible vapor pressure, high thermal stability, and tunable physicochemical properties, ILs exhibit remarkable tolerance to HTHS conditions compared to conventional surfactants and chemicals [31,32,33,34]. Their applications have expanded to include roles as demulsifiers [35,36], catalysts, and, pertinently, as agents for enhanced oil recovery [37,38,39]. ILs can significantly reduce interfacial tension (IFT) and alter rock wettability, thereby mobilizing capillary-trapped residual oil [37].

The integration of ILs with polymers represents a paradigm shift toward multi-functional, hybrid CEOR formulations [40,41]. While some molecular-level synergies (e.g., rheology modification, competitive adsorption) have been proposed [42,43], a robust and practical implementation is the sequential injection strategy adopted in this study: an IL pre-flush alters rock wettability and reduces residual oil saturation, creating a conditioned zone for a subsequent polymer chase that provides macroscopic mobility control. This process-level synergy leverages the distinct advantages of each agent while avoiding the complexity of in situ chemical interactions.

The design and optimization of such complex processes rely heavily on reservoir simulation. Core-flooding simulation, in particular, serves as a critical bridge between laboratory experiments and field-scale prediction [44,45]. Modern simulators incorporate sophisticated models for polymer behavior, including shear-dependent viscosity, adsorption isotherms, permeability reduction, and IPV [46]. History-matching core-flood experiments allows for the calibration of these key parameters, transforming a qualitative understanding into a quantitative and predictive model. This validated model is indispensable for scaling up laboratory results, forecasting field performance, optimizing operational parameters like slug sizes and concentrations, and ultimately de-risking the substantial investment required for a full-field CEOR project [47,48]. Without this rigorous numerical validation, the extrapolation of core-scale results remains highly uncertain.

The use of chemical EOR in HTHS reservoirs faces complex challenges as mentioned previously, primarily related to the stability of the injection fluids and their economic feasibility. Acrylamido-tertiary-butyl sulfonate (ATBS) copolymers are currently considered as the industry benchmark for thermal stability. A comprehensive review by Seright and Wang confirmed that ATBS provides superior resistance to hydrolysis compared to HPAM [49]. However, utilization of ATBS is not without significant limitations. A critical 2024 study by Sebastian et al. revealed that ATBS polymer retention in carbonate reservoirs is highly sensitive to water salinity; retention values were found to double in high-salinity seawater compared to diluted make-up water, raising concerns about the economic feasibility of chemical banks in harsh marine environments [50]. Furthermore, injectivity issues persist in low-permeability carbonates (<100 mD). Mushtaq et al. showed that despite their thermal robustness, ATBS polymers tend to filtrate and degrade mechanically in tight pore throats, which necessitates rigorous pre-shearing protocols that complicate field operations [51].

Parallel attentions have concentrated on hydrophobically associating polymers (HAPs) and bio-based derivatives. Recent works by Liu et al. and Yi et al. highlight the ability of HAPs to enhance viscosity through intermolecular aggregation networks, which are efficient in offshore conditions like the Bohai oilfield [52,53]. Similarly, eco-friendly alternatives such as hydrophobically modified chitosan have been discovered for their dual ability to increase viscosity and alter wettability [54]. However, the synthesis of these smart polymers is complex, and their solubility is often lacking in very high-salinity brines, resulting in potential phase separation or precipitation [55,56].

To overcome the precipitation issues of anionic surfactants in high-salinity brines, betaine-based zwitterionic surfactants have gained significance. Recently, Deng et al. successfully synthesized local zwitterionic surfactants that were stable and altered wettability in both sandstone and carbonate formations without the precipitation typical of anionic chemicals [57]. More recently, studies have presented novel lignin-based and oleic-acid-based zwitterionic formulations capable of significant viscosity reduction in heavy oil [58,59,60]. Additionally, zwitterionic polymers (zPAM) have been developed to withstand high shear rates, showing better rheological performance than standard HPAM [61].

Despite these chemical successes, major operational challenges were identified in a key recent study by Alvarenga et al. regarding topside processing. The study found that residual zwitterionic surfactants in produced fluids tend to stabilize water-in-crude oil emulsions, significantly inhibiting the water–oilseparation process and increasing the costs of demulsification [62]. Moreover, adsorption remains a problem; Golab stated that the adsorption of zwitterionic polymeric surfactants on sandstone increases linearly with salinity, potentially leading to excessive chemical loss in HTHS reservoirs [63].

Nanotechnology represents a hot topic in EOR studies, with extensive research lately confirming the effectiveness of nanoparticles (silica, TiO_2_, Al_2_O_3_) in altering wettability and reducing IFT [64,65,66]. The most recent experimental studies have shifted towards hybrid nanofluids, such as surface-modified silica or MoS_2_ combined with surfactants. Wen et al. and Tliba et al. proved that these hybrids offer synergistic effects, improving oil recovery beyond what is possible with standalone fluids [67,68].

However, the transition from lab to field is hindered by stability challenges. As noted by Rizvi and Tong et al., nanofluids struggle with long-term dispersion stability in high-salinity environments, where particle agglomeration can cause severe pore plugging [69]. Although polymer-coated nanoparticles show improved transport [70,71], the cost of functionalization and the complexity of preparing stable nanofluids on a field scale remain prohibitive for many projects [72].

As shown in Table 1, the proposed hybrid IL–HPAM system offers a practical and economically viable alternative to emerging high-temperature, high-salinity (HTHS) EOR technologies by directly addressing their key limitations:

Cost and Practicality: It replaces costly ATBS copolymers—whose adsorption doubles in high-salinity brines—with standard HPAM injected in diluted formation brine (20% salinity). The IL pre-flush chemically conditions the rock (reducing residual oil saturation via wettability alteration and IFT reduction), which mitigates HPAM retention and enables effective mobility control under salinity conditions that would otherwise degrade unmodified polymers—eliminating the need for expensive sulfonated alternatives.

Operational Complexity: It avoids the topside separation complications caused by zwitterionic surfactants, which stabilize water-in-oil emulsions and increase operational costs.

Stability and Scalability: It circumvents the long-term dispersion instability and high cost of nanofluid hybrids, which suffer from agglomeration in saline environments.

Mechanistic Integration: It combines wettability alteration/IFT reduction (via the IL) and mobility control (via HPAM) into a single, sequential injection process, eliminating the need for complex multi-component formulations.

This literature analysis reveals a clear gap: ATBS polymers suffer from high retention costs; zwitterionic systems complicate downstream separation; and nanofluids face stability and scalability hurdles.

Furthermore, field applications of polymer flooding have demonstrated the importance of proper design based on comprehensive reservoir characterization [73]. The success of any chemical EOR project depends on understanding reservoir heterogeneity, which affects polymer placement and sweep efficiency [74]. Numerical simulation provides the framework to integrate geological data, fluid properties, and chemical behavior to create realistic predictions of field performance [75,76].

Despite compelling experimental evidence for IL–HPAM synergy from core-flood studies [77,78,79], a significant methodological gap persists: a dedicated numerical study that (i) builds a rigorously calibrated simulation framework from such data, (ii) performs systematic optimization of the hybrid process, (iii) quantitatively deconvolutes the underlying displacement mechanisms, and (iv) assesses robustness in geologically heterogeneous systems—a critical step toward field-scale deployment—has not been presented. This work fills these gaps by developing a history-matched core-scale model, identifying an optimal injection strategy, validating the dual-displacement mechanism, and demonstrating scalability through 3D heterogeneous simulation. This approach transforms discrete experimental results into a predictive, optimization-ready framework for HTHS reservoirs.

## 2. Materials and Methods

### 2.1. Materials

Crude Oil: Saudi medium crude oil was used as the oleic phase. Its physical properties, measured at 23 °C, are listed in Table 2.

**Figure 1:**
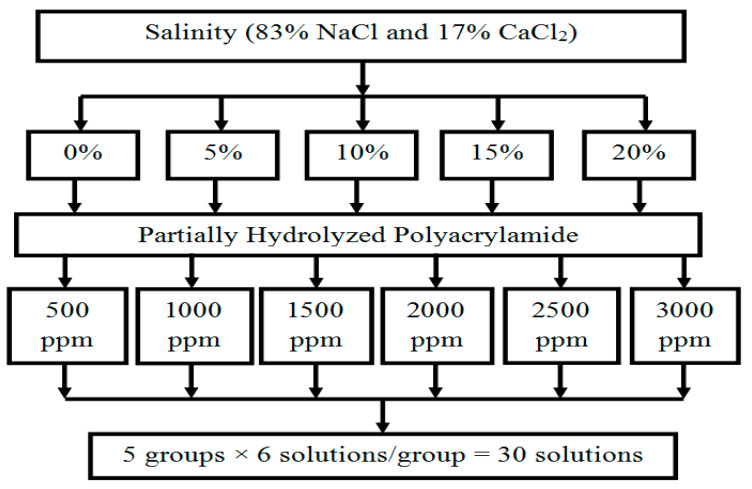
Preparation of 30 different HPAM polymer solutions used for viscosity measurements at various concentrations, salinities, and temperatures.

Brine and Chemicals: Brines were prepared with salinities of 0, 5, 10, 15, and 20% total dissolved solids (TDS), with a weight ratio of 83% NaCl to 17% CaCl_2_. The polymer used in this study was partially hydrolyzed polyacrylamide (HPAM) and the ionic liquid was Ammoeng 102. A total of 30 polymer solutions were prepared for viscosity measurements, as illustrated in Figure 1.

Core Samples: Fifteen core samples of Berea sandstone were used. Table 3 summarizes their dimensions and petrophysical properties.

### 2.2. Experimental Setup and Procedure

Viscosity Measurements: The viscosities of all fluids were measured using a Brookfield DV-II+ Pro Viscometer at various shear rates and temperatures (60 °C to 90 °C).

**Figure 2:**
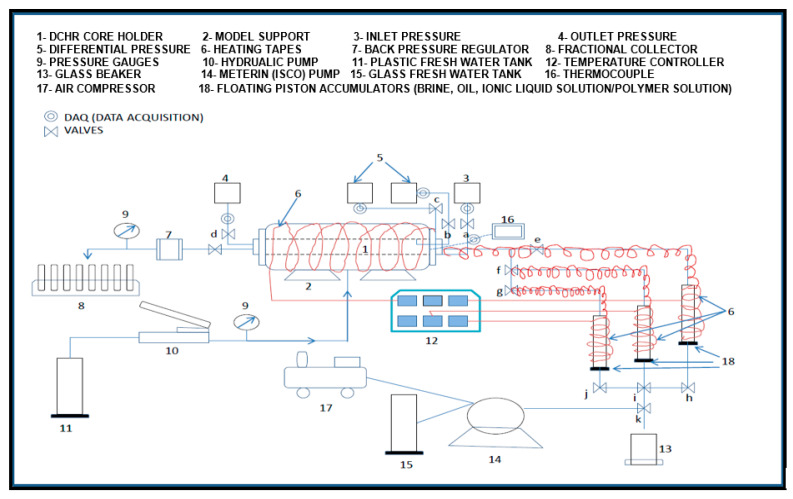
Schematic diagram of the core-flooding system CFS-200 [79].

**Figure 3:**
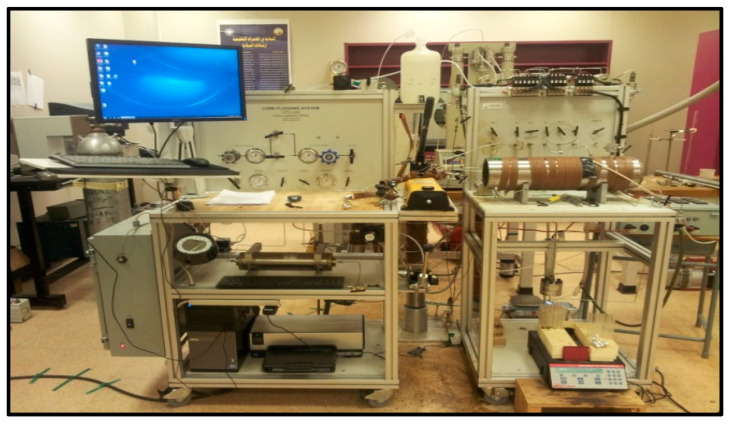
Physical setup of the core-flooding system CFS-200.

Core-flooding System: The core-flooding system (CFS-200) was used for all experiments. The schematic and physical setup are shown in Figure 2 and Figure 3, respectively.

Experimental Plan: The flooding sequence of ionic liquid and polymer buffer was conducted in secondary mode at reservoir conditions of 5000 psi confining pressure and 2000 psi pore pressure. A summary of all flooding runs is presented in Table 4.

### 2.3. Numerical Simulation Methodology

**Figure 4:**
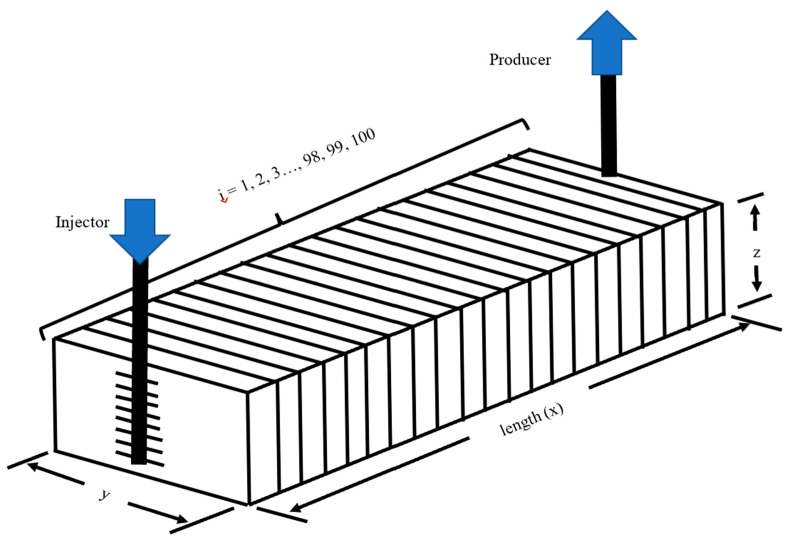
Schematic of the 1D homogeneous simulation model showing grid configuration and flow geometry.

The experimental core-floods were simulated using the commercial reservoir simulator Schlumberger ECLIPSE 100. To represent the cylindrical core geometry within the simulator’s Cartesian framework, a one-dimensional (1D) equivalent grid system (100 × 1 × 1) was constructed, preserving pore volume, cross-sectional area, and length to maintain hydraulic equivalence. Figure 4 represents the 1D homogeneous simulation model used for calibration, consisting of 100 grid cells aligned along the core length (x-direction), with an injector at cell i = 1 and a producer at cell i = 100. Flow is strictly linear, replicating the experimental core flood geometry. Y- and Z-dimensions are collapsed to a single grid block each (100 × 1 × 1), preserving hydraulic equivalence while enabling focused calibration of chemical displacement mechanisms.

A two-stage simulation workflow was adopted to ensure model robustness and scalability:

Stage 1 (Calibration): A 1D homogeneous model was used to replicate the linear flow geometry of the laboratory core flood. The simulation explicitly includes three sequential phases: (i) an initial water injection stage representing the ionic liquid (IL) pre-flush, (ii) a polymer chase with HPAM, and (iii) a final water chase. Consistent with the objective of isolating polymer behavior, only parameters governing polymer transport and rock interactions were calibrated; all rock and fluid properties were fixed to experimentally measured values.

Stage 2 (Validation): The calibrated parameters from Stage 1 were transferred without modification to a three-dimensional (3D), three-layer heterogeneous model (100 × 1 × 3 grid) featuring a five-fold vertical permeability contrast. This step tested the predictive capability of the model under geologically stratified conditions.

Polymer flooding was simulated using the standard ECLIPSE 100 polymer module, which accounts for:

Polymer Rheology: Aqueous-phase viscosity enhancement as a function of concentration, implemented using the experimentally measured PLYVISC relationship (no further tuning was applied).

Polymer–Rock Interactions: Adsorption, resistance factor (RF), residual resistance factor (RRF), and inaccessible pore volume (IPV), modeled via the PLYADS and PLYROCK keywords.

History-Matching Philosophy and Fixed Parameters: All rock and fluid properties were held fixed at independently measured or literature-based values:

Porosity: 0.193

Permeability: 209 mD (homogeneous base case)

Initial Water and Oil Saturation: 0.30 and 0.70

PVT and Density: From laboratory measurements

Relative Permeability: Water–oil relative permeability curves were defined with a reduced residual oil saturation of $S_{or}=0.23$ for the entire simulation. This value was determined experimentally after IL treatment and represents a reduction from the pre-flood baseline of $S_{or}=0.32$ observed in conventional high-salinity waterfloods. The reduction is supported by complementary evidence of wettability alteration and interfacial tension reduction. This parameter was held fixed and not adjusted during history matching.

Calibration Scope: Only the following polymer-specific transport parameters were calibrated: Polymer adsorption isotherm (PLYADS), resistance and residual resistance factors (PLYROCK), and polymer viscosity parameters (PLYVISC).

Simulation Injection Strategy: The schedule mirrors the experimental protocol:

First 0.627 h: Water injection at 15 SCC/hour, representing the IL pre-flush stage. The experimentally measured $S_{or}=0.23$ embedded in the relative permeability curves captures its net effect on displacement efficiency.

Next 0.564 h: HPAM injection at 500 ppm (20.9 kg/m^3^).

Final 4.9 h: Water chase to monitor tail-end production.

Cumulative oil recovery (FOE) is reported relative to original oil in place (OOIP), based on an initial oil saturation of 0.70. Thus, FOE includes oil produced during both the IL-mimicking waterflood and the polymer chase stages, enabling direct comparison with total experimental recovery. The use of the post-IL $S_{or}=0.23$ (versus the baseline 0.32) ensures the model accurately reflects the enhanced displacement efficiency of the hybrid IL-HPAM process.

Validation in a Heterogeneous 3D Model: The calibrated polymer transport parameters were applied directly to the 3D layered model without adjustment. The model reproduced realistic production dynamics and sweep behavior, confirming that the parameter set represents a physically consistent description of in situ polymer performance, not an artifact of overfitting, and remains valid under conditions of increased geological complexity. This issue is discussed later in Section 3.

## 3. Results and Discussion

### 3.1. Optimization of Ionic Liquid Slug Size

**Figure 5:**
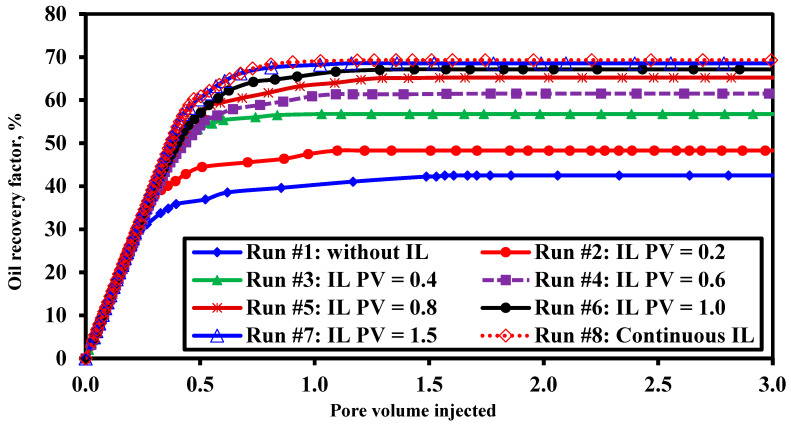
Oil recovery factor versus cumulative injected PV with varying IL slug sizes.

**Figure 6:**
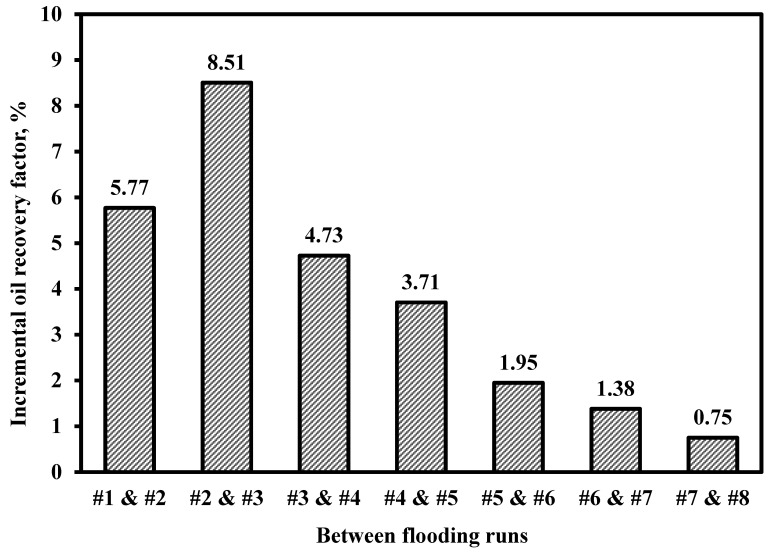
Incremental oil recovery factor gained between consecutive flooding runs for IL slug optimization.

The relationship between injected ionic liquid (IL) slug size and ultimate oil recovery was investigated. The results demonstrate that oil recovery increased with the volume of the IL slug; however, the incremental gain diminished significantly beyond 0.8 pore volumes (PV). The recovery factor rose markedly from 42.5% for the brine flood to 56.8% with a 0.4 PV IL slug. Although continuous IL injection achieved the highest recovery (69.3%), the marginal improvement beyond 0.8 PV was not economically justifiable. Therefore, a 0.4 PV IL slug was selected as the optimal size for all subsequent hybrid floods, offering a substantial recovery increase with efficient chemical utilization. Table 5 and Figure 5 summarize the effect of pore volume of the injected ionic liquid while Figure 6 summarizes the effect of increasing ionic liquid solution slug size on oil recovery.

### 3.2. Synergistic Effect of Polymer Buffer on IL Flooding

**Figure 7:**
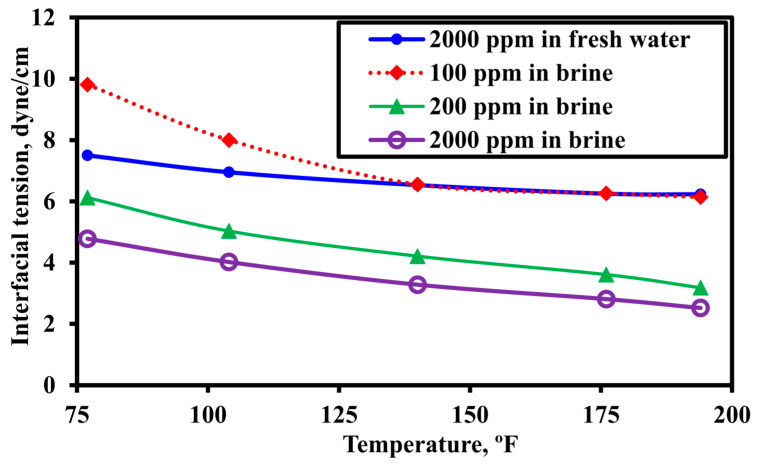
Interfacial tension as a function of temperature for Ammoeng 102 in water and 10 wt% NaCl brine (data from Benzagouta [80]).

Figure 7 illustrates the systematic reduction in interfacial tension (IFT) with increasing ionic liquid concentration under isothermal conditions. Notably, the presence of brine substantially enhanced interfacial activity, yielding significantly lower IFT values than those measured in deionized water at equivalent concentrations and temperatures. Across all systems evaluated, Ammoeng 102 exhibited the lowest IFT, with its performance further improving as temperature increased.

**Figure 8:**
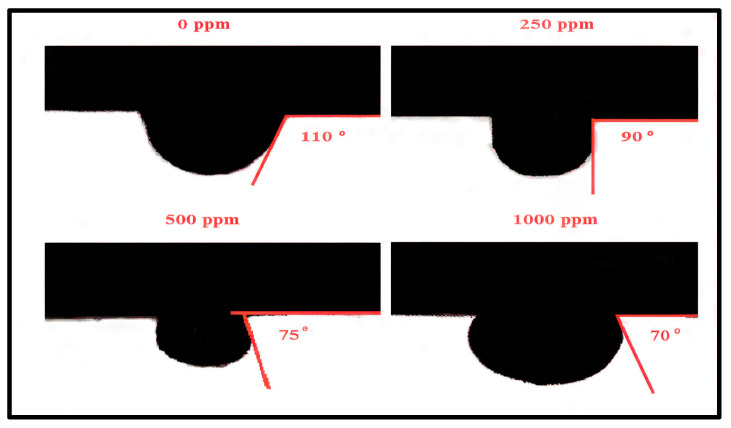
Contact angle of oil droplet with rock surface in presence of different concentrations of Ammoeng 102 IL solution (data from Bin Dahbag et al. [81]).

To quantify the wettability alteration induced by the ionic liquid (IL), advancing contact angle measurements were conducted on Berea sandstone surfaces saturated with Saudi medium crude oil in brine solutions containing varying concentrations of Ammoeng 102. As shown in Figure 8, the contact angle decreased systematically from 110° in the absence of IL—indicative of a strongly oil-wet surface—to 90° at 250 ppm, 75° at 500 ppm, and finally 70° at 1000 ppm. This progressive reduction, spanning a total decrease of 40°, reflects a clear transition toward an intermediate water-wet state. The trend demonstrates that the IL effectively adsorbs onto the rock surface, modifying its wettability and thereby reducing the capillary forces responsible for residual oil trapping. Consequently, the observed behavior provides direct experimental support for the proposed mechanism of enhanced microscopic displacement efficiency through IL-induced wettability alteration.

**Figure 9:**
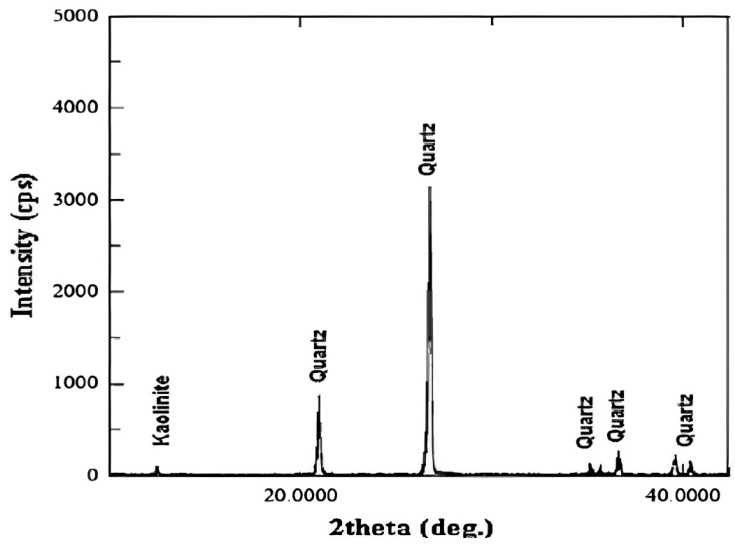
X-ray diffraction (XRD) pattern of the Berea sandstone core sample.

The XRD analysis shown in Figure 9 confirms that the Berea sandstone is predominantly quartz, with kaolinite as the only detectable clay mineral. The pattern is dominated by sharp, high-intensity quartz reflections, whereas kaolinite appears only as minor, low-intensity peaks. Semi-quantitative assessment based on the overwhelming relative peak intensities indicates that quartz constitutes more than 90% of the crystalline phases.

**Figure 10:**
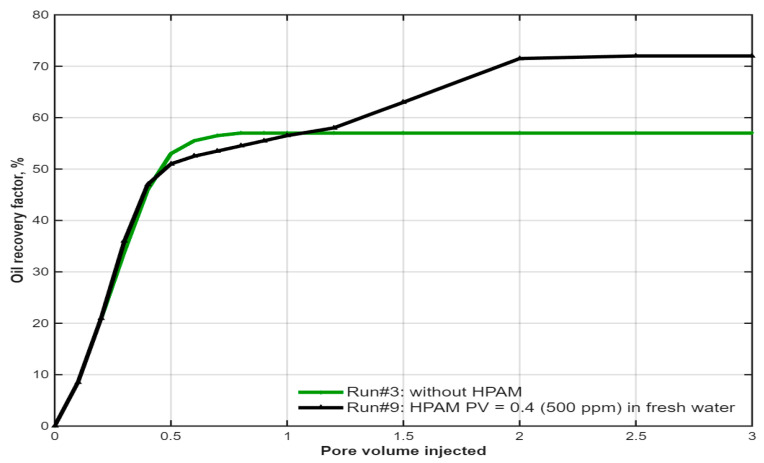
Comparison of oil recovery profiles for an IL flood (Run #3) and the IL-HPAM system (Run #9). Reproduced from [Omer et.al.], arcjournals.org, 2017 [79].

The core objective of this study was to evaluate the impact of a polymer buffer injected after the optimized IL slug. A comparison of the IL-only flood and the hybrid flood (IL followed by a 0.4 PV polymer slug in diluted formation brine (20% salinity, ~50,000 ppm TDS)) revealed a distinct three-stage recovery profile, as shown in Figure 10. In the first stage, where the IL slug was being injected in both runs, the recovery profiles were nearly identical. The second stage commenced with the injection of the polymer slug, which caused a temporary lag in recovery. This is attributed to the transition to a lower-salinity environment during polymer injection, which temporarily reduces the effectiveness of residual IL in further lowering interfacial tension, as the IL pre-flush was optimized under higher-salinity conditions. In the third and final stage, the high viscosity of the polymer solution began to dominate the displacement process. The improved mobility ratio led to the mobilization of additional oil, resulting in a higher ultimate recovery [82]. This profile clearly demonstrates the successful synergy between the IL, which reduces residual oil saturation, and the polymer, which enhances macroscopic sweep efficiency.

(Note: The HPAM solution was prepared using distilled water as a base but diluted with formation brine to 20% salinity prior to injection, ensuring compatibility with high-salinity reservoir conditions.)

### 3.3. Effect of Polymer Solution Salinity

**Figure 11:**
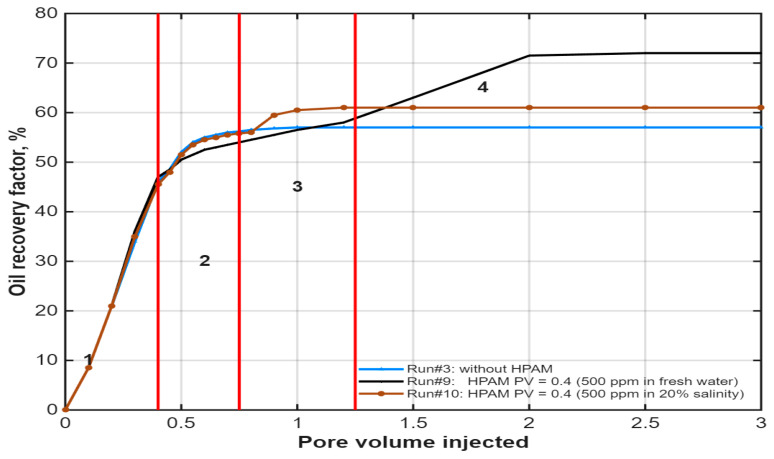
Effect of polymer drive salinity on oil recovery for the hybrid IL-HPAM system. Reproduced from [Omer et.al.], arcjournals.org, 2017 [79].

The critical role of polymer drive salinity was investigated by comparing a 20%-salinity polymer buffer (Run #9, 500 ppm HPAM in diluted formation brine) with a full-salinity one (Run #10, 500 ppm HPAM in 100% formation brine), as shown in Figure 11. The comparison reveals a nuanced performance profile shaped by competing salinity-dependent mechanisms.

With the full-salinity polymer (Run #10), the recovery profile closely paralleled that of the IL-only flood (Run #3) during the initial and middle displacement stages. This suggests that the elevated salinity helped maintain the IL’s interfacial activity and wettability-alteration effectiveness. However, the high ionic strength significantly reduced the polymer’s viscosity, leading to a poorer mobility ratio and diminished sweep efficiency in the tertiary stage. Consequently, the full-salinity case achieved a final recovery of approximately 62.6%—only marginally higher than the IL-only case (~60.6%).

In contrast, the 20%-salinity polymer buffer (Run #9) exhibited a slightly delayed response but ultimately delivered superior mobility control and higher recovery (~70% range based on trend). Although the lower salinity may have temporarily diluted the in situ salinity—potentially causing a brief performance dip—it enabled much greater polymer viscosity development. This resulted in better frontal stability, improved macroscopic sweep, and higher ultimate displacement efficiency.

This comparison highlights a critical operational trade-off: while higher salinity helps preserve IL effectiveness, it severely compromises polymer viscosity and mobility control. The results demonstrate that a moderate-salinity polymer drive (20% formation brine), despite potential transient dilution effects, enables superior conformance and higher ultimate recovery—a finding with direct implications for designing hybrid IL–polymer formulations in high-salinity reservoirs.

### 3.4. Effect of Polymer Slug Size and Concentration

**Figure 12:**
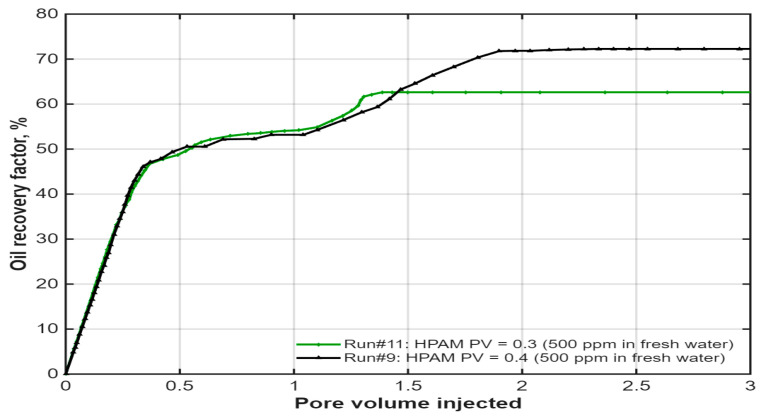
Effect of polymer slug size on oil recovery in the hybrid IL-HPAM system. Reproduced from [Omer et.al.], arcjournals.org, 2017 [79].

Effect of Slug Size: The influence of polymer volume was examined by comparing a 0.4 PV polymer slug (Run #9) with a 0.3 PV slug (Run #11), as illustrated in Figure 12. The results show that the reduced slug size yielded a lower ultimate recovery (~64.72% vs. ~60.5%, estimated from trend). The 0.3 PV slug was insufficient to sustain the favorable mobility ratio long enough to displace the same volume of oil as the 0.4 PV slug. This confirms that a sufficiently large polymer slug is critical to maximizing sweep efficiency in the hybrid process.

**Figure 13:**
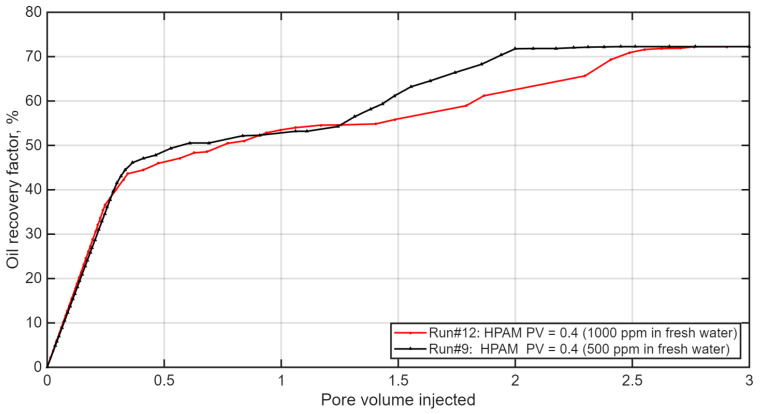
Effect of polymer concentration on oil recovery for the hybrid IL-HPAM system. Reproduced from [Omer et.al.], arcjournals.org, 2017 [79].

Effect of Polymer Concentration: The impact of polymer concentration was evaluated by comparing 500 ppm (Run #9) and 1000 ppm (Run #12) solutions, as shown in Figure 13. Contrary to the expectation of diminishing returns, the higher concentration provided both greater viscosity and a significantly improved ultimate recovery, increasing from approximately 64.7% to 72.7%. This indicates that while 500 ppm offers an effective balance between mobility control and chemical usage, further viscosity enhancement from 1000 ppm can substantially boost displacement efficiency in this system, likely through improved frontal stability and more effective suppression of viscous fingering.

Process-Level Synergy: The observed performance enhancement arises from a sequential, process-level synergy rather than molecular-scale interactions. The IL (Ammoeng 102) first alters rock wettability and reduces interfacial tension, mobilizing residual oil into an “oil bank”. The subsequent HPAM slug then provides high-viscosity drive and permeability reduction, stabilizing the front and sweeping the mobilized oil toward the producer. This complementary action—where the IL acts as a preconditioning agent and the HPAM as a sweep-enhancement agent—results in recovery greater than the sum of individual effects, as validated by history-matched simulation.

### 3.5. Effect of Temperature

**Figure 14:**
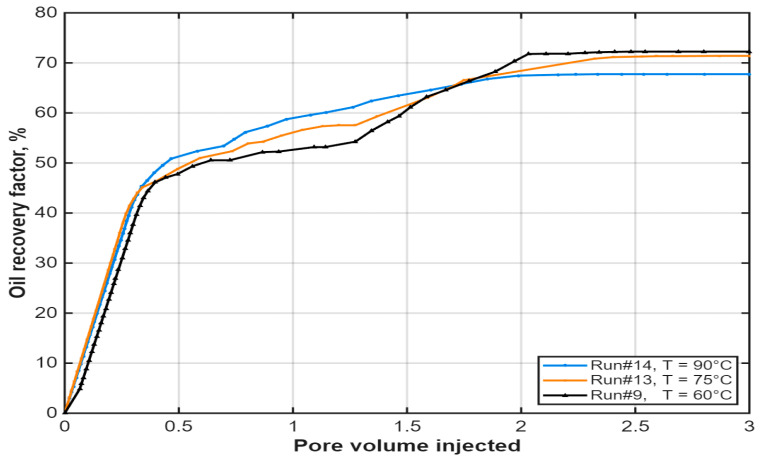
Influence of temperature on oil recovery profile of the hybrid IL-HPAM system at PV = 0.4. Reproduced from [Omer et.al.], arcjournals.org, 2017 [79].

The efficiency of the hybrid process at elevated temperatures was investigated at 60 °C, 75 °C, and 90 °C. The results (Figure 14) showed that while breakthrough occurred at the same injected volume (PV = 0.4) across all temperatures, higher temperatures led to a more rapid decline in polymer performance post-breakthrough. Although elevated temperature reduces the viscosity of both crude oil and polymer solution, the net effect under tested conditions accelerated the thermal degradation of HPAM, resulting in diminished viscosity retention and lower displacement efficiency at 75 °C and 90 °C compared to 60 °C.

### 3.6. Effect of Injection Rate

**Figure 15:**
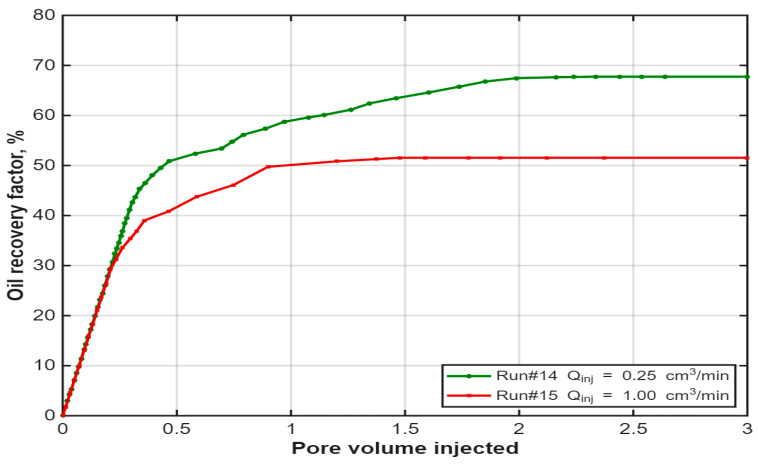
Impact of injection rate on oil recovery of the hybrid IL-HPAM system. Reproduced from [Omer et.al.], arcjournals.org, 2017 [79].

The impact of injection rate was evident when comparing low (0.25 cm^3^/min) and high (1.00 cm^3^/min) rates, as shown in Figure 15. The four-fold increase in injection rate drastically reduced the recovery factor from 67.7% to 51.5%. The higher rate induced early breakthrough of the chemical slugs, promoted viscous fingering and oil bypassing and likely caused mechanical degradation of the polymer molecules, thereby reducing its effective viscosity. This underscores the importance of employing a low, controlled injection rate to maximize contact time and volumetric sweep efficiency in chemical EOR processes.

### 3.7. Mechanistic Interpretation and History Matching

**Figure 16:**
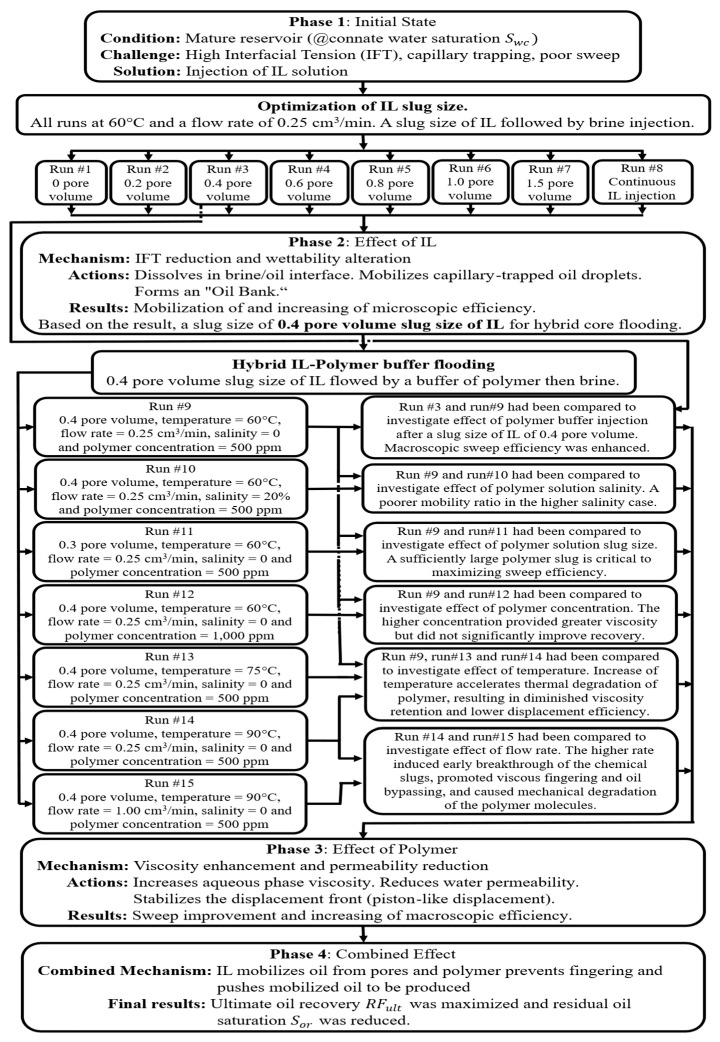
Schematic flowchart of the hybrid IL-HPAM flooding process.

The exceptional performance of the hybrid ionic liquid–polymer flood is rooted in a sequential synergy between two distinct mechanisms: the ionic liquid first mobilizes residual oil by reducing interfacial tension and altering wettability, while the subsequent HPAM polymer slug improves displacement efficiency through enhanced mobility control and in-depth flow diversion. As detailed in Figure 16, this schematic provides a comprehensive visual overview of the sequential mechanisms and experimental optimization of a hybrid ionic liquid (IL) and polymer (HPAM) flooding process. The process begins with the injection of an IL slug into a mature reservoir at connate water saturation, where high interfacial tension (IFT) and capillary trapping limit sweep efficiency. The IL reduces IFT and alters wettability, mobilizing trapped oil into an “oil bank” and thereby enhancing microscopic displacement efficiency. Experimental optimization identified a 0.4 pore volume (PV) IL slug as optimal under the given conditions. This is followed by a polymer buffer, which increases aqueous-phase viscosity and reduces water permeability to stabilize the displacement front and improve macroscopic sweep efficiency. Parametric studies revealed that polymer solution salinity and slug size critically influence mobility control, while elevated temperature and injection rates can accelerate polymer degradation and promote viscous fingering. Ultimately, the schematic illustrates the synergistic mechanism by which the IL mobilizes capillary-trapped oil and the polymer provides the viscous drive to maximize ultimate oil recovery and minimize residual oil saturation.

The polymer exhibits a favorable combination of transport properties—high viscosity enhancement, low adsorption, deep propagation, and significant permeability reduction—that enable it to act as an effective “mobility-control buffer” following the initial mobilization phase.

A critical characteristic is its low adsorption capacity, reaching saturation at only 0.01 kg/kg-rock, which minimizes irreversible chemical loss and allows the polymer to penetrate deeply into the reservoir core. Furthermore, the polymer demonstrates a substantial capacity for permeability reduction, as indicated by a residual resistance factor (RRF) of 2.633 (Table 5). This value corresponds to an approximately 62% reduction in water permeability, which actively contributes to in-depth flow diversion and improved conformance control by blocking swept, high-permeability pathways and diverting the chemical slug to unswept zones. This indicates that the recovery mechanism relies on a powerful combination of viscosity-driven mobility control and significant permeability modification, as quantified by the parameters summarized in Table 5.

Complementing this is the polymer’s ability to increase aqueous phase viscosity by a factor of 4.5–5.0 at 0.4–0.5 kg/m^3^ (500 ppm), significantly improving the mobility ratio, suppressing viscous fingering, and stabilizing the displacement front during the tertiary stage. Additionally, the polymer experiences inaccessible pore volume (IPV) effects, where its large macromolecules are excluded from approximately 8% of the pore space, causing the polymer front to advance slightly faster than a tracer—a well-documented phenomenon in polymer flooding.

The synergy is defined as a sequential ‘mobilize-and-sweep’ effect. The IL (Ammoeng 102) first acts as a preconditioning agent: its amphiphilic structure leads to drastic IFT reduction (Figure 7) and wettability alteration to water-wet conditions (Figure 8), thereby mobilizing residual oil. The subsequent HPAM slug then acts as a sweep-enhancement agent: it provides viscosity for mobility control and, through in situ adsorption, creates permeability reduction for flow diversion (Table 5), efficiently banking and recovering the mobilized oil. This complementary action—where the IL alters the micro-scale environment to free oil and the HPAM improves the macro-scale flow to capture it, results in recovery greater than the sum of individual effects, as validated by the history-matched simulation.

These characteristics explain the system’s sensitivity to operational conditions. As shown in Figure 14, increasing temperature from 60 °C (Run #9) to 90 °C (Run #14) leads to progressive degradation of HPAM stability. At higher temperatures, thermal and hydrolytic breakdown reduce the polymer’s molecular weight and effectiveness, resulting in a more rapid decline in response after breakthrough (PV = 0.4). This underscores the importance of moderate reservoir temperatures for maintaining polymer integrity.

Similarly, injection rate plays a crucial role, as demonstrated in Figure 15, which compares Run #14 (0.25 cm^3^/min) and Run #15 (1.00 cm^3^/min). Despite identical polymer concentration, Run #14 achieves significantly higher ultimate recovery than Run #15. The lower rate allows for better frontal stability, longer contact time, and reduced shear stress, minimizing mechanical degradation and improving sweep efficiency. In contrast, the high injection rate promotes early post-breakthrough inefficiency and less effective displacement, highlighting the advantage of controlled, low-rate operation.

**Figure 17:**
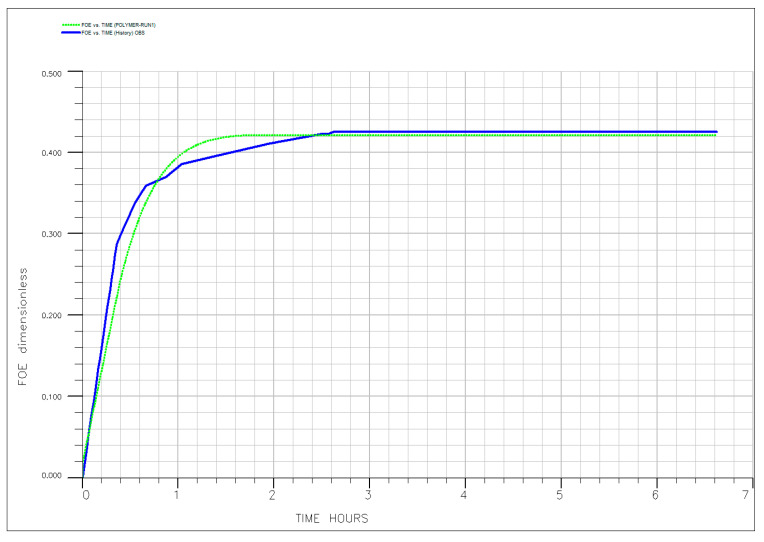
History matching of the experimental (solid line) and simulated (dotted line) oil recovery profile for core flooding for Run #1.

**Figure 18:**
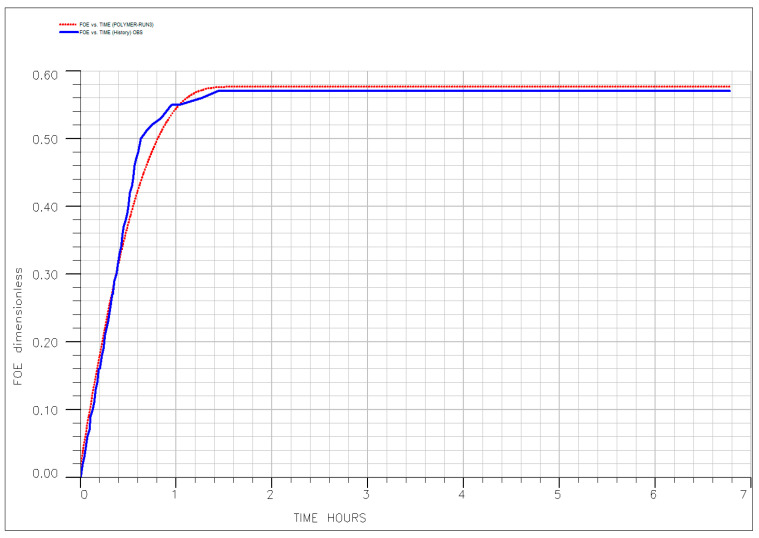
History matching of the experimental (solid line) and simulated (dotted line) oil recovery profile for core flooding for Run #3.

**Figure 19:**
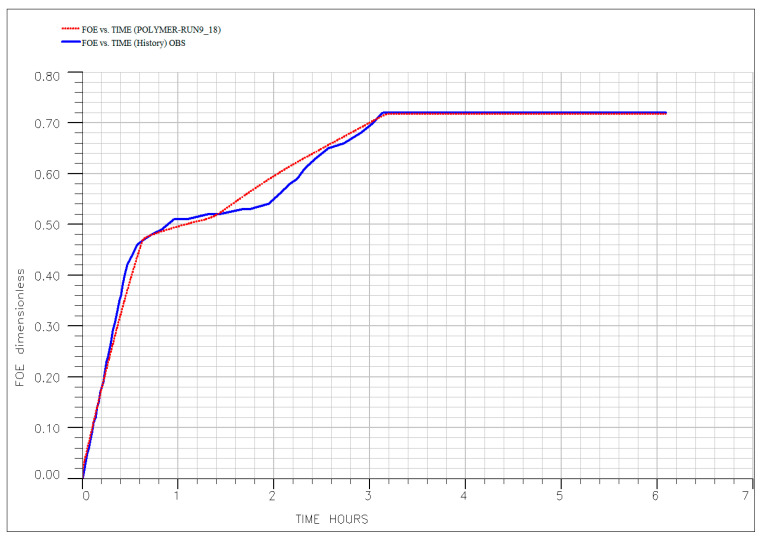
History matching of the experimental (solid line) and simulated (dotted line) oil recovery profile for core flooding for Run #9.

**Figure 20:**
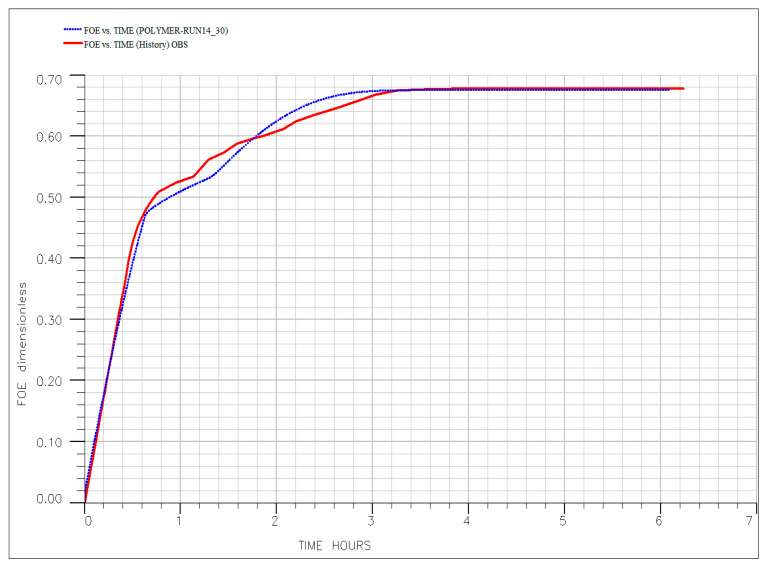
History matching of the experimental (solid line) and simulated (dotted line) oil recovery profile for core flooding for Run #14.

**Figure 21:**
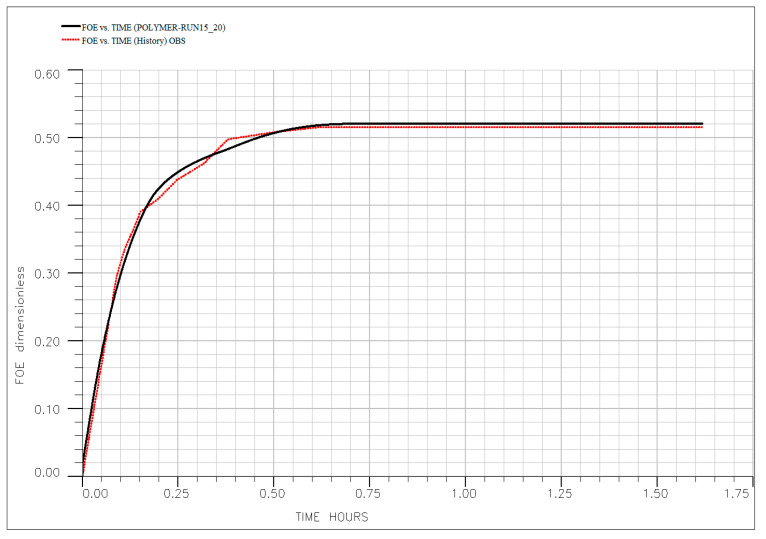
History matching of the experimental (solid line) and simulated (dotted line) oil recovery profile for core flooding for Run #15.

To quantitatively validate the experimental findings and the interpreted mechanisms, a comprehensive numerical history-matching exercise was conducted. The simulation model, incorporating the history-matched polymer transport parameters summarized in Table 6, successfully reproduced the oil recovery profiles across all key experimental runs—as illustrated in Figure 17, Figure 18, Figure 19, Figure 20 and Figure 21. The close agreement between simulated (dotted line) and experimental (solid line) results provides a high degree of confidence in the calibrated model. Specifically, the model accurately captures the characteristic three-stage recovery profile: initial mobilization by the ionic liquid; a transient lag phase associated with transition to diluted brine and equilibration; and sustained viscous-dominated displacement by the polymer front. This successful history matching validates not only the numerical representation of polymer transport and rheology but also the fundamental premise of the sequential synergy: the ionic liquid first mobilizes trapped oil, and the polymer recovers it via a stabilized, high-viscosity drive supported by measurable permeability modification. The calibrated model thus establishes a robust framework for scaling up and optimizing this hybrid EOR process for field-level application, significantly de-risking future implementation. Optimal performance requires moderate temperatures (<80 °C) and low injection rates to preserve polymer integrity, ensure adequate adsorption development, and maintain both mobility control and flow diversion. For reservoirs exceeding these limits, alternative polymers with superior thermal or shear stability (e.g., ATBS-based copolymers) should be considered. Meanwhile, economic optimization suggests that moderate concentrations (~500 ppm) balance technical performance with cost-effectiveness, avoiding unnecessary chemical expenditure while maximizing recovery.

The injection of diluted formation brine (20% salinity) into Berea sandstone carries a known risk of inducing clay swelling and fines migration. In our system, the substantial measured RRF of 2.633 is interpreted to be primarily a consequence of in situ polymer adsorption and hydrodynamic retention. This conclusion is quantitatively supported by our simulation study: the numerical model achieved an excellent history match of the oil recovery and pressure data across all experiments by calibrating only polymer transport and rheological parameters (Table 6, Figure 16, Figure 17, Figure 18, Figure 19 and Figure 20). The model did not require, and the data did not exhibit, a signature necessitating the inclusion of a separate, dynamic permeability damage function to account for clay effects. This indicates that any formation damage signal was minor relative to the dominant effect of the polymer and thus did not materially impact the overall recovery mechanism or the calibration of our predictive model.

### 3.8. Robustness of the Optimized IL-HPAM Process to Stratified Permeability Variation

**Figure 22:**
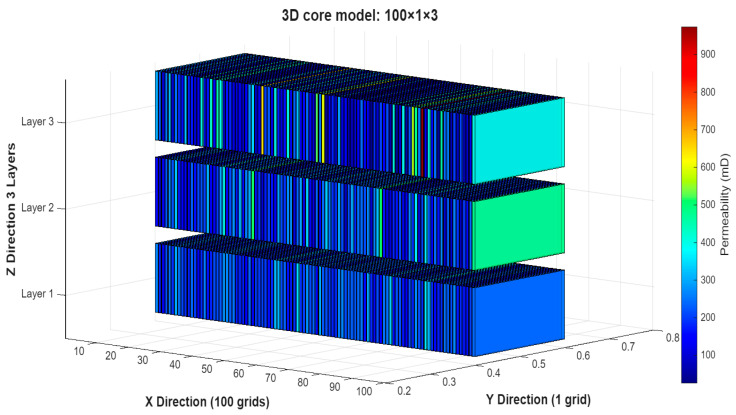
Three-dimensional layered simulation model showing permeability distribution for the heterogeneous case.

To evaluate the robustness of the optimized hybrid IL–HPAM process under geologically realistic conditions, a sensitivity study was conducted using a three-dimensional (3D), three-layer simulation model (grid dimensions: 100 × 1 × 3), as illustrated in Figure 22. Base petrophysical properties—including porosity, pore volume, and initial saturations—were derived from Core #14 (see Table 2), with an areal average permeability of kavg = 209 mD. Two permeability distributions were considered:

Homogeneous: A uniform permeability of 209 mD assigned to all layers.

**Figure 23:**
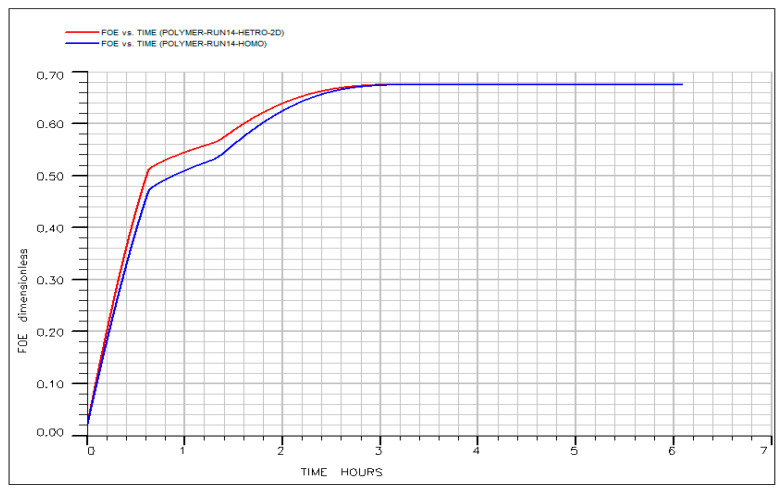
Oil recovery vs. time for homogenous and heterogeneous system.

**Figure 24:**
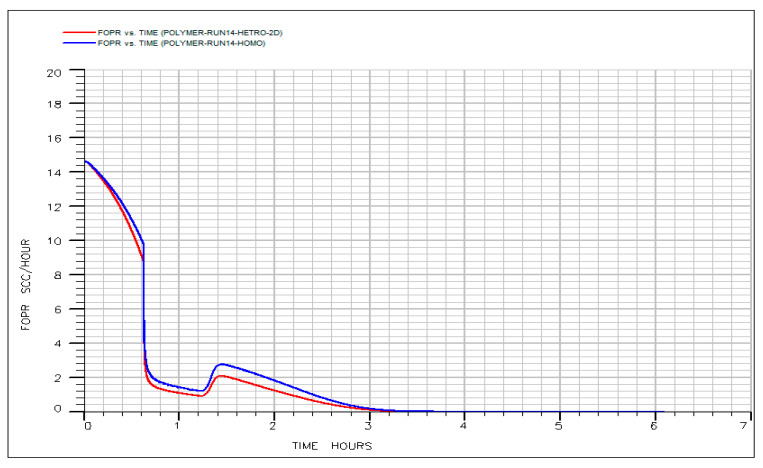
Oil production vs. time for homogenous and heterogeneous system.

Heterogeneous: A five-fold permeability contrast applied across the layers (e.g., a high-permeability middle layer flanked by lower-permeability top and bottom layers), while preserving the same areal average permeability of 209 mD. This configuration represents typical stratified reservoir heterogeneity. Both models were subjected to identical optimal chemical design (0.4 PV IL + 0.4 PV HPAM at 500 ppm in diluted formation brine (20% salinity)) and injection protocol. The resulting oil production rate and oil recovery profiles are shown in Figure 23 and Figure 24. The comparison reveals that layered heterogeneity had only a minimal impact on overall performance. The heterogeneous case exhibited a marginally higher initial flow rate (~15 vs. 14.5 SCC/h), consistent with accelerated early drainage through the high-permeability layer. However, this difference was transient; both oil rate curves converged within approximately 2 h, indicating rapid re-establishment of displacement stability. Most significantly, ultimate recovery was nearly identical: the homogeneous model achieved a final oil recovery of ~0.690, compared to ~0.685 for the heterogeneous case, a difference of less than 1% of original oil in place (OOIP). This negligible gap demonstrates that the adverse effects typically associated with moderate permeability contrasts, such as early breakthrough and volumetric bypass, were effectively mitigated by the designed chemical sequence.

These results confirm that the tailored HPAM slug in diluted formation brine (20% salinity) provides sufficient viscosity and mobility control to maintain efficient sweep despite vertical permeability variation. Consequently, the optimized hybrid IL–HPAM process exhibits strong robustness to stratified heterogeneity, a critical attribute for reliable field-scale deployment in layered reservoirs. This finding further validates the predictive capability of our simulation-based optimization framework and underscores its utility in de-risking hybrid chemical EOR applications in geologically complex settings.

## 4. Conclusions

This study presents a rigorously calibrated numerical framework that validates and optimizes a hybrid IL–HPAM flooding process for high-salinity, high-temperature reservoirs. Key findings include:

(i) The identification of an optimal injection sequence—0.4 PV of Ammoeng 102 IL followed by 0.4 PV of HPAM (500 ppm) in diluted formation brine (20% salinity)—which delivers up to 15% OOIP incremental recovery over IL flooding alone;
(ii) Clear mechanistic deconvolution confirming a dual displacement process: IL-induced microscopic oil mobilization (via wettability alteration and IFT reduction) followed by HPAM-driven macroscopic sweep improvement (via mobility control and flow diversion);
(iii) Robust performance in a 3D heterogeneous layered model, demonstrating scalability and predictive capability for field-scale application.

Nevertheless, this work has limitations. First, the polymer transport parameters (adsorption, residual resistance factor, and inaccessible pore volume) were calibrated to match experimental recovery data rather than independently measured through static adsorption isotherms or tracer tests. Second, the viscosity model relies on bulk-phase rheology and does not explicitly account for mechanical degradation under porous media shear conditions. Third, the use of Berea sandstone—a quartz-dominated, non-swelling rock—may not reflect challenges in formations with reactive clays or carbonates.

Future work should focus on: (i) conducting in situ polymer transport experiments under reservoir-relevant shear and salinity conditions to constrain model parameters; (ii) extending the simulation framework to more complex lithologies, including clay-rich or fractured systems; and (iii) advancing toward a field pilot to validate the economic and operational viability of the hybrid IL–HPAM process in real-world HTHS reservoirs.

## Figures and Tables

**Table 1:** Comparative analysis of the proposed hybrid IL-HPAM system versus state-of-the-art EOR technologies for HTHS reservoirs.

EOR Technology	Primary Mechanism	Key Advantages (HTHS)	Critical Limitations and Challenges	Key References
ATBS copolymers	Steric hindrance and charge repulsion (sulfonate groups)	Industry standard for high thermal stability (>100 °C) and shear resistance.	High retention cost: adsorption doubles in high-salinity seawater compared to diluted brine, threatening project economics. Injectivity: prone to filtration issues in tight carbonates (<100 mD).	Seright and Wang (2023) [49]; Sebastian et al. (2024) [50]; Mushtaq et al. (2021) [51].
Hydrophobically associating polymers (HAPs)	Associative intermolecular networking	Enhanced viscosity at lower concentrations; suited for offshore conditions (e.g., Bohai field).	Solubility sensitivity: complex synthesis; prone to phase separation or precipitation in hyper-saline brines if not perfectly tuned.	Liu et al. (2025) [52]; Yi et al. (2022) [53]; Afolabi et al. (2019) [56].
Zwitterionic surfactants	IFT reduction and wettability alteration	Excellent solubility in high salinity; noprecipitation (unlike anionic surfactants).	Topside separation issues: residual surfactants stabilize water-in-oil emulsions, complicating dehydration and increasing OPEX. Adsorption: adsorption on sandstone increases linearly with salinity.	Deng et al. (2024) [57]; Alvarenga et al. (2025) [62]; Golab (2025) [63].
Nanofluid hybrids (e.g., SiO_2_, MoS_2_)	Disjoining pressure and surface modification	Synergistic IFT reduction; wettability alteration to water-wet.	Stability and scalability: long-term dispersion stability is difficult in high salinity (agglomeration risks); functionalized nanofluids are expensive.	Wen et al. (2025) [67]; Rizvi (2024) [65]; Tong et al. (2023) [69].
Hybrid IL–HPAM (this work)	Dual-displacement process: (1) IL-induced wettability alteration and IFT reduction → microscopic oil mobilization; (2) HPAM-driven mobility control → macroscopic sweep improvement	Cost and Simplicity: Uses standard HPAM (no expensive ATBS); avoids emulsion issues (vs. zwitterionics); scalable (vs. nanofluids). Salinity Management: IL pre-flush enables effective HPAM performance in diluted high-salinity brine (20% formation salinity).	Process Optimization: Requires precise design of IL slug size, HPAM concentration, and salinity zoning.	Current study

**Table 2:** Crude oil physical properties at 23 °C and asphaltene content analysis.

Physical Property	Value
Specific gravity	0.885
Gravity, °API	28.37
Density, g/cm^3^	0.883
Viscosity, cp	23.0
Asphaltene content, %	9.60 *
Asphaltene carbon content, %	81.29 *
Asphaltene hydrogen content, %	9.13 *
Asphaltene nitrogen content, %	0.70 *
Asphaltene other elements content %	8.88 *

* Bin Dahbag [78].

**Table 3:** Dimensions and petrophysical properties of the Berea Sandstone samples.

Run No.	Diameter, cm	Length, cm	Bulk Volume, cm^3^	Dry Weight, gm	Saturated Weight, gm	Pore Volume, cm^3^	Porosity, %	Absolute Permeability, md
#1	3.78	11.53	129.34	274.0	302.9	25.08	19.39	246
#2	3.78	11.44	128.33	273.8	302.8	25.16	19.61	243
#3	3.78	11.82	132.65	282.0	311.7	25.77	19.43	233
#4	3.78	12.04	135.06	284.8	315.9	26.98	19.98	240
#5	3.78	11.69	131.13	281.8	312.1	26.29	20.05	217
#6	3.79	10.82	122.07	257.3	284.7	23.77	19.48	215
#7	3.79	9.90	111.69	235.9	260.8	21.61	19.34	225
#8	3.79	11.56	130.42	275.4	304.4	25.16	19.29	204
#9	3.84	10.72	124.15	258.2	285.3	23.51	18.94	221
#10	3.80	10.25	115.89	243.4	269.1	22.30	19.24	216
#11	3.85	11.26	131.03	271.8	301.6	25.86	19.73	202
#12	3.78	11.83	132.76	279.7	310.1	26.38	19.87	210
#13	3.69	12.03	128.60	259.7	287.6	24.21	18.82	211
#14	3.80	11.45	129.52	273.3	301.6	24.56	18.96	209
#15	3.81	11.31	128.61	282.0	311.2	25.34	19.70	212

**Table 4:** Summary of core-flooding runs.

Run No.	Q, cm^3^/min	T, °F (°C)	IL Solution PV	HPAM Solution PV	PC, ppm	HPAM Solution Salinity, %
#01	0.25	140 (60)	0.0	20% salinity brine was injected directly after ionic liquid solution without polymer solution injection.		
#02	0.25	140 (60)	0.2			
#03	0.25	140 (60)	0.4			
#04	0.25	140 (60)	0.6			
#05	0.25	140 (60)	0.8			
#06	0.25	140 (60)	1.0			
#07	0.25	140 (60)	1.5			
#08	0.25	140 (60)	3.9 *			
#09	0.25	140 (60)	0.4	0.4	500	0
#10	0.25	140 (60)	0.4	0.4	500	20
#11	0.25	140 (60)	0.4	0.3	500	0
#12	0.25	140 (60)	0.4	0.4	1000	0
#13	0.25	167 (75)	0.4	0.4	500	0
#14	0.25	194 (90)	0.4	0.4	500	0
#15	1.00	194 (90)	0.4	0.4	500	0

* Continuous ionic liquid flooding from the start to the end without 20% salinity brine injection.

**Table 5:** Ionic liquid core-flooding runs summary.

Run No.	Injected Ionic Liquid Slug Size/PV	Connate Water Saturation (S_wc_), %	Residual Oil Saturation (S_or_), %	Ultimate Oil Recovery (RF_ult_), %
#1	0.0	25.42	42.87	42.52
#2	0.2	27.07	37.72	48.29
#3	0.4	27.70	31.24	56.79
#4	0.6	22.47	29.83	61.52
#5	0.8	25.18	26.02	65.23
#6	1.0	25.67	24.40	67.18
#7	1.5	26.54	23.10	68.56
#8	3.9	25.80	22.77	69.31

**Table 6:** History-matched polymer transport parameters used in the simulation.

Parameter	Unit	Optimum/Used Value	Role in Recovery Process
Injection concentration	kg/m^3^	0.4–0.5	Balances viscosity gain with chemical cost.
Viscosity multiplier	-	4.5–5.0 (at 0.4–0.5 kg/m^3^)	Primary driver for mobility control and sweep improvement.
Max. adsorption capacity	kg/kg-rock	0.012 (0.5 kg/m^3^)	Defines chemical loss; low value aids deep propagation.
Residual resistance factor	-	2.633	Indicates minimal permeability reduction; favors injectivity over diversion.
Inaccessible pore volume	fraction	0.08	Causes faster polymer front velocity, improving economic efficiency.

## Data Availability

All data supporting the findings of this work are presented within the article. For any further inquiries, please contact the corresponding author.

## References

[1] Green D.W., Willhite G.P. (2018). Enhanced Oil Recovery.

[2] Ahmed T. (2018). Reservoir Engineering Handbook.

[3] Lake L.W., Johns R., Rossen B., Pope G.A. (2014). Fundamentals of Enhanced Oil Recovery.

[4] Hernández F.A.T., Moreno R.B.Z.L. (2020). The effectiveness of computed tomography for the experimental assessment of surfactant-polymer flooding. Oil Gas Sci. Technol.—Rev. D’ifp Energ. Nouv..

[5] Sheng J.J. (2010). Modern Chemical Enhanced Oil Recovery: Theory and Practice.

[6] Wever D.A.Z., Picchioni F., Broekhuis A.A. (2011). Polymers for enhanced oil recovery: A paradigm for structure–property relationship in aqueous solution. Prog. Polym. Sci..

[7] Seright R.S.S. (2010). Potential for polymer flooding reservoirs with viscous oils. SPE Reserv. Eval. Eng..

[8] Hematpour H., Mardi M., Edalatkhah S., Arabjamaloei R. (2011). Experimental study of polymer flooding in low-viscosity oil using one-quarter five-spot glass micromodel. Pet. Sci. Technol..

[9] Sochi T. (2010). Flow of non-Newtonian fluids in porous media. J. Polym. Sci. B Polym. Phys..

[10] Samanta A., Bera A., Ojha K., Mandal A. (2010). Effects of alkali, salts, and surfactant on rheological behavior of partially hydrolyzed polyacrylamide solutions. J. Chem. Eng. Data.

[11] Zhang G., Seright R.S.S. (2014). Effect of concentration on HPAM retention in porous media. SPE J..

[12] Al-Hajri S., Mahmood S.M., Abdulelah H., Akbari S. (2018). An overview on polymer retention in porous media. Energies.

[13] Sorbie K.S. (2013). Polymer-Improved Oil Recovery.

[14] Dawson R., Lantz R.B. (1972). Inaccessible pore volume in polymer flooding. Soc. Pet. Eng. J..

[15] Delshad M., Kim D.H., Magbagbeola O.A., Huh C., Pope G.A., Tarahhom F. (2008). Mechanistic interpretation and utilization of viscoelastic behavior of polymer solutions for improved polymer-flood efficiency. Proceedings of the SPE Improved Oil Recovery Conference.

[16] Zhang F., Jiang Y., Liu P., Wang B., Sun S., Hua D., Zhao J. (2022). Laboratory experimental study on polymer flooding in high-temperature and high-salinity heavy oil reservoir. Appl. Sci..

[17] Kamal M.S., Sultan A., Hussein I., Hussain S.M., AlSofi A.M. (2018). Screening of surfactants and polymers for high temperature high salinity carbonate reservoirs. Proceedings of the SPE Kingdom of Saudi Arabia Annual Technical Symposium and Exhibition.

[18] Gbadamosi A., Patil S., Kamal M., Adewunmi A., Adeyinka Y., Agi A., Oseh J. (2022). Application of polymers for Chemical Enhanced Oil Recovery: A review. Polymers.

[19] Muller G. (1981). Thermal stability of high-molecular-weight polyacrylamide aqueous solutions. Polym. Bull..

[20] Zaitoun A., Potie B. (1983). Limiting conditions for the use of hydrolyzed polyacrylamides in brines containing divalent ions. Proceedings of the SPE International Conference on Oilfield Chemistry.

[21] Abidin A.Z., Puspasari T., Nugroho W.A. (2012). Polymers for enhanced oil recovery technology. Procedia Chem..

[22] Welton T. (1999). Room-temperature ionic liquids. Solvents for synthesis and catalysis. Chem. Rev..

[23] Rogers R.D., Seddon K.R. (2003). Ionic liquids-solvents of the future?. Science.

[24] Plechkova N.V., Seddon K.R. (2008). Applications of ionic liquids in the chemical industry. Chem. Soc. Rev..

[25] Hallett J.P., Welton T. (2011). Room-temperature ionic liquids: Solvents for synthesis and catalysis. 2. Chem. Rev..

[26] Domańska U. (2005). Solubilities and thermophysical properties of ionic liquids. Pure Appl. Chem..

[27] Nandwani S.K., Chakraborty M., Bart H.-J., Gupta S. (2018). Synergism, phase behaviour and characterization of ionic liquid-nonionic surfactant mixture in high salinity environment of oil reservoirs. Fuel.

[28] da Silva E.B., Santos D., de Brito M.P., Guimarães R.C.L., Ferreira B.M.S., Freitas L.S., de Campos M.C.V., Franceschi E., Dariva C., Santos A.F. (2014). Microwave demulsification of heavy crude oil emulsions: Analysis of acid species recovered in the aqueous phase. Fuel.

[29] Hazrati N., Beigi A.A.M., Abdouss M. (2018). Demulsification of water in crude oil emulsion using long chain imidazolium ionic liquids and optimization of parameters. Fuel.

[30] Bin-Dahbag M.S., Al Quraishi A.A., Benzagouta M.S., Kinawy M.M., Al Nashef I.M., Al Mushaegeh E. (2014). Experimental study of use of ionic liquids in enhanced oil recovery. J. Pet. Environ. Biotechnol..

[31] Hanamertani A.S., Pilus R.M., Irawan S. (2017). A review on the application of ionic liquids for enhanced oil recovery. ICIPEG 2016: Proceedings of the International Conference on Integrated Petroleum Engineering and Geosciences.

[32] RSaw K., Pillai P., Mandal A. (2022). Synergistic effect of low saline ion tuned Sea Water with ionic liquids for enhanced oil recovery from carbonate reservoirs. J. Mol. Liq..

[33] Nagy R., Hartyányi M., Bejczi R., Bartha L., Puskás S. (2025). Recent aspects of chemical enhanced oil recovery. Chem. Pap..

[34] Esfandiarian A., Maghsoudian A., Shirazi M., Tamsilian Y., Kord S., Sheng J.J. (2021). Mechanistic investigation of the synergy of a wide range of salinities and ionic liquids for enhanced oil recovery: Fluid–fluid interactions. Energy Fuels.

[35] Liang K., Han P., Chen Q., Su X., Feng Y. (2019). Comparative study on enhancing oil recovery under high temperature and high salinity: Polysaccharides versus synthetic polymer. ACS Omega.

[36] Pal N., Saxena N., Laxmi K.V.D., Mandal A. (2018). Interfacial behaviour, wettability alteration and emulsification characteristics of a novel surfactant: Implications for enhanced oil recovery. Chem. Eng. Sci..

[37] TChávez-Miyauchi E., Kar T., Firoozabadi A. (2025). Synergy of Polymer Mobility Control and Surfactant for Interface Elasticity Increase in Improved Oil Recovery. SPE J..

[38] Musa M.S.M., Agi A., Nwaichi P.I., Ridzuan N., Mahat S.Q.B. (2023). Simulation study of polymer flooding performance: Effect of salinity, polymer concentration in the Malay Basin. Geoenergy Sci. Eng..

[39] Schlumberger (2021). ECLIPSE Technical Description.

[40] Liu P., Zhang K., Yao J. (2023). Reservoir automatic history matching: Methods, challenges, and future directions. Adv. Geo-Energy Res..

[41] BinDahbag M.S., Hassanzadeh H., AlQuraishi A.A., Benzagouta M.S. (2019). Suitability of ionic solutions as a chemical substance for chemical enhanced oil recovery—A simulation study. Fuel.

[42] Levitt D.B., Pope G.A. (2008). Selection and screening of polymers for enhanced-oil recovery. Proceedings of the SPE Improved Oil Recovery Conference.

[43] Vermolen E.C., Van Haasterecht M.J., Masalmeh S.K., Faber M.J., Boersma D.M., Gruenenfelder M. (2011). Pushing the envelope for polymer flooding towards high-temperature and high-salinity reservoirs with polyacrylamide based ter-polymers. Proceedings of the SPE Middle East Oil and Gas Show and Conference.

[44] Khamis M.A., Omer O.A., Kinawy M.M. (2018). Predicting the optimum concentration of partially hydrolyzed polyacrylamide polymer in brine solutions for better oil recovery, experimental study. Proceedings of the SPE Kingdom of Saudi Arabia Annual Technical Symposium and Exhibition.

[45] Zhang G., Seright R.S. (2015). Hydrodynamic retention and rheology of EOR polymers in porous media. Proceedings of the SPE International Conference on Oilfield Chemistry.

[46] Lai N., Wen Y., Yang Z., Chen J., Zhao X., Wang D., He W., Chen Y. (2020). Polymer flooding in high-temperature and high-salinity heterogeneous reservoir by using diutan gum. J. Pet. Sci. Eng..

[47] Rock A., Hincapie R.E., Tahir M., Langanke N., Ganzer L. (2020). On the role of polymer viscoelasticity in enhanced oil recovery: Extensive laboratory data and review. Polymers.

[48] Song K., Tao J., Lyu X., Xu Y., Liu S., Wang Z., Liu H., Zhang Y., Fu H., Meng E. (2022). Recent advances in polymer flooding in China. Molecules.

[49] Seright R.S., Wang D. (2023). Polymer flooding: Current status and future directions. Pet. Sci..

[50] Sebastian A., Mushtaq M., Al-Shalabi E.W., AlAmeri W., Mohanty K., Masalmeh S., AlSumaiti A.M. (2024). Investigating the effects of make-up water dilution and oil presence on polymer retention in carbonate reservoirs. Sci. Rep..

[51] Mushtaq M., Alfazazi U., Thomas N.C., Al-Shalabi E.W., AlAmeri W., Masalmeh S., AlSumaiti A. (2025). ATBS Polymer Injectivity in 22–86 md Carbonate Cores: Impacts of Polymer Filtration, Mechanical Shearing, and Oil Presence. SPE J..

[52] Liu T., Chen X., Tang X. (2025). Reservoir Compatibility and Enhanced Oil Recovery of Polymer and Polymer/Surfactant System: Effects of Molecular Weight and Hydrophobic Association. Polymers.

[53] Yi F., Huang B., Wang C., Tang X., Wang X., Liu Q., Su Y., Chen S., Wu X., Chen B. (2022). Hydrophobically associating polymers dissolved in seawater for enhanced oil recovery of Bohai offshore oilfields. Molecules.

[54] Scerbacova A., Sharfan I.I.B., Abdulhamid M.A. (2025). Hydrophobically Modified Chitosan-Based Polymers for Enhanced Oil Recovery. CleanMat.

[55] Bhut P.R., Pal N., Mandal A. (2019). Characterization of hydrophobically modified polyacrylamide in mixed polymer-gemini surfactant systems for enhanced oil recovery application. ACS Omega.

[56] Afolabi R.O., Oluyemi G.F., Officer S., Ugwu J.O. (2019). Hydrophobically associating polymers for enhanced oil recovery–Part A: A review on the effects of some key reservoir conditions. J. Pet. Sci. Eng..

[57] Deng X., Alotaibi M.B., Fahmi M., Patil S., Mahmoud M., Kamal M.S., Hussain S.M.S. (2024). Locally synthesized zwitterionic surfactants as EOR chemicals in sandstone and carbonate. ACS Omega.

[58] Wu Q., Liu T., Xu X., Yang J. (2025). A Lignin-Based Zwitterionic Surfactant Facilitates Heavy Oil Viscosity Reduction via Interfacial Modification and Molecular Aggregation Disruption in High-Salinity Reservoirs. Molecules.

[59] Wang W., Liang M.-Y., Lang J.-Q., Mtui H.I., Yang S.-Z., Mu B.-Z. (2024). A new thermal-tolerant bio-based zwitterionic surfactant for enhanced oil recovery. Green Mater..

[60] Wang W., Liang M.-Y., Lang J.-Q., Mtui H.I., Yang S.-Z., Mu B.-Z. (2024). A new bio-based zwitterionic surfactant with improved interfacial activity by optimizing hydrophilic head. Biomass Convers. Biorefin..

[61] Karimov D., Imekova G., Toktarbay Z., Nuraje N. (2025). Experimental and numerical simulation studies of the zwitterionic polymers for enhanced oil recovery. Sci. Rep..

[62] Alvarenga B.G., Duncke A.C.P., Pérez-Gramatges A., Percebom A.M. (2025). Impact of Residual Zwitterionic Surfactants on Topside Water–Oil Separation of Pre-Salt Light Crude Oil Emulsions. ACS Omega.

[63] Golab E.G. (2025). Evaluation of zwitterionic and polymeric surfactant adsorption for enhanced oil recovery in sandstone reservoirs with high salinity conditions. J. Pet. Explor. Prod. Technol..

[64] Akpan U.E. (2024). Nanotechnology in Enhanced oil Recovery: A Review of Current Research. Chem. Sci. Int. J..

[65] Hassan R.S.M. (2024). Nanotechnology Applications in Enhanced Oil Recovery (EOR). Int. J. Sci. Res. Manag..

[66] El-Masry J.F., Bou-Hamdan K.F., Abbas A.H., Martyushev D.A. (2023). A comprehensive review on utilizing nanomaterials in enhanced oil recovery applications. Energies.

[67] Wen Y., Zhang C., Zhu G., Wang J., Taqi A.A.M., Liang T. (2025). Combined Effects of Nanofluid and Surfactant on Enhanced Oil Recovery: An Experimental Study. ACS Omega.

[68] Tliba L., Sagala F., Hethnawi A., Glover P.W.J., Menzel R., Hassanpour A. (2024). Surface-modified silica nanoparticles for enhanced oil recovery in sandstone cores. J. Mol. Liq..

[69] Tong Q., Fan Z., Liu Q., Qiao S., Cai L., Fu Y., Zhang X., Sun A. (2023). Research progress in nanofluid-enhanced oil recovery technology and mechanism. Molecules.

[70] Bila A., Stensen J.Å., Torsæter O. (2019). Experimental investigation of polymer-coated silica nanoparticles for enhanced oil recovery. Nanomaterials.

[71] Bila A., Stensen J.Å., Torsæter O. Polymer-functionalized silica nanoparticles for improving water flood sweep efficiency in Berea sandstones. Proceedings of the 2019 International Symposium of the Society of Core Analysts (SCA 2019).

[72] Goharzadeh A., Fatt Y.Y., Sangwai J.S. (2023). Effect of TiO_2_–SiO_2_ hybrid nanofluids on enhanced oil recovery process under different wettability conditions. Capillarity.

[73] Xin X., Yu G., Wu K., Dong X., Chen Z. (2021). Polymer flooding in heterogeneous heavy oil reservoirs: Experimental and simulation studies. Polymers.

[74] Meybodi H.E., Kharrat R., Ghazanfari M.H. (2008). Effect of heterogeneity of layered reservoirs on polymer flooding: An experimental approach using 5-spot glass micromodel. Proceedings of the SPE Europec Featured at EAGE Conference and Exhibition.

[75] Computer Modelling Group Ltd (2023). STARS User Guide.

[76] Todd M.R., Longstaff W.J. (1972). The development, testing, and application of a numerical simulator for predicting miscible flood performance. J. Pet. Technol..

[77] Dyes A.B., Caudle B.H., Erickson R.A. (1954). Oil production after breakthrough as influenced by mobility ratio. J. Pet. Technol..

[78] Dahbag M.S.B. (2014). Effect of Ionic Liquids on the Efficiency of Crude Oil Recovery.

[79] Omer O., Kinawy M., Khamis M. (2017). Effect of Using Polymer Buffer on Efficiency of Crude Oil Recovery by Ionic Liquids. Int. J. Pet. Petrochem. Eng..

[80] Benzagouta M.S., AlNashef I.M., Karnanda W., Al-Khidir K. (2013). Ionic liquids as novel surfactants for potential use in enhanced oil recovery. Korean J. Chem. Eng..

[81] Dahbag M.S.B., Hossain M.E., AlQuraishi A.A. (2016). Efficiency of Ionic Liquids as an Enhanced Oil Recovery Chemical: Simulation Approach. Energy Fuels.

[82] Sakthivel S., Velusamy S., Nair V.C., Sharma T., Sangwai J.S. (2017). Interfacial tension of crude oil-water system with imidazolium and lactam-based ionic liquids and their evaluation for enhanced oil recovery under high saline environment. Fuel.

